# The Association between Obesity and Chronic Conditions: Results from a Large Electronic Health Records System in Saudi Arabia

**DOI:** 10.3390/ijerph182312361

**Published:** 2021-11-24

**Authors:** Suliman Alghnam, Saleh A. Alessy, Mohamed Bosaad, Sarah Alzahrani, Ibrahim I. Al Alwan, Ali Alqarni, Riyadh Alshammari, Mohammed Al Dubayee, Majid Alfadhel

**Affiliations:** 1 Population Health Section-King Abdullah International Medical Research Centre (KAIMRC), King Saud Bin Abdulaziz University for Health Sciences (KSAU-HS), Riyadh 11426, Saudi Arabia; sarahabdullatifz@gmail.com; 2 Public Health Department, College of Health Sciences, Saudi Electronic University, Riyadh 11673, Saudi Arabia; s.alessy@seu.edu.sa; 3 Department of Epidemiology, School of Public Health, University of Pittsburg, Pittsburg, PA 15261, USA; MAB530@pitt.edu; 4 Department of Pediatrics, Division of Endocrinology, King Abdulaziz Medical City, King Abdullah Specialist Children’s Hospital, Ministry of National Guard-Health Affairs, Riyadh 11426, Saudi Arabia; profialalwan@gmail.com (I.I.A.A.); aldubayeemo@NGHA.MED.SA (M.A.D.); 5 College of Medicine, King Abdulaziz Medical City, King Saud Bin Abdulaziz University for Health Sciences, Ministry of National Guard-Health Affairs, Riyadh 11426, Saudi Arabia; 6 King Abdullah International Medical Research Centre, King Abdulaziz Medical City, King Saud Bin Abdulaziz University for Health Sciences (KSAU-HS), Ministry of National Guard-Health Affairs, Alahsa 11426, Saudi Arabia; QarniAA@ngha.med.sa; 7 School of Public Health, King Saud Bin Abdulaziz University for Health Sciences (KSAU-HS), Riyadh 11426, Saudi Arabia; riyadalshammari@gmail.com; 8 Medical Genomic Research Department, King Abdullah International Medical Research Centre, King Abdulaziz Medical City, Ministry of National Guard-Health Affairs, Riyadh 11426, Saudi Arabia; dralfadhelm@gmail.com; 9 Genetics and Precision Medicine Department (GPM), King Abdullah Specialist Children’s Hospital, King Abdulaziz Medical City, Ministry of National Guard-Health Affairs, Riyadh 11426, Saudi Arabia; 10 King Abdulaziz Medical City, King Saud Bin Abdulaziz University for Health Sciences, Ministry of National Guard-Health Affairs, Riyadh 11426, Saudi Arabia

**Keywords:** obesity, diabetes, electronic health record, hypertension, Saudi Arabia

## Abstract

This cross-sectional study aimed to estimate the prevalence of obesity and its association with diabetes and hypertension among beneficiaries in the National Guard Health Affairs system of Saudi Arabia. We included individuals aged 17 years and older, and patients were classified as diabetic or hypertensive if they had any visit during the 4 years where the primary diagnosis was one of those conditions or they were taking diabetes or hypertension medications. The association between obesity (body mass index ≥30) and diabetes and hypertension were evaluated using a multiple logistic regression model, adjusting for age, gender, nationality, and region. A total of 616,092 individuals were included. The majority were Saudi nationals (93.1%). Approximately 68% of the population were either obese (38.9%) or overweight (29.30%). Obesity was more prevalent among Saudi nationals (39.8% vs. 26.7%, *p* < 0.01) and females (45.3% vs. 31.2%, *p* < 0.01). Obesity was independently associated with diabetes mellitus (OR = 2.24, *p* < 0.01) and hypertension (OR = 2.15, *p* < 0.01). The prevalence of obesity in the study population was alarming and more pronounced among women. Our findings call for efforts to intensify preventive measures to reduce obesity and associated conditions. Using electronic records to examine the impact of interventions to reduce obesity and chronic conditions may help monitor and improve population health.

## 1. Introduction

Obesity is a critical global public health concern and has become an epidemic in many countries. The World Health Organization (WHO) estimates that obesity rates have almost tripled since 1975 [1]. While as many as 108 million people in the world faced severe food insecurity in 2016, there were more than 1.9 billion overweight adults worldwide (39%) [2]. Of these adults, more than 650 million were obese, representing 13% of all adults worldwide [1,3]. In almost all countries, the concurrent acceleration in obesity prevalence appears to be motivated primarily by shifts in the global food system, which produces more refined, inexpensive, and efficiently prepared food than ever before. This passive energy overconsumption that leads to obesity is a predictable consequence of market economies focused on consumption-based growth [4].

In the Gulf Cooperation Council (GCC) countries, the incidence of obesity has risen dramatically over the past few decades (Kuwait, Saudi Arabia, Oman, United Arab Emirates, Qatar, and Bahrain). This increase was driven by rapid economic development and prosperity following oil discovery in the region [3]. Factors, such as urbanization, restricted access to walkable areas, sedentary lifestyles, hot weather, changes in food choices, and car-dependent cities switched the leading causes of death from infectious to non-communicable diseases (NCDs) in the GCC countries including Saudi Arabia [3]. These factors also constitute significant drivers of obesity in SA. Previously published nationwide surveys showed an increase of about 74% in the prevalence of obesity in SA [5]. This progressive increase led to an increase in obesity from 22% in 1990 to 36% in 2005 [6,7]. However, according to the WHO, adult obesity decreased slightly to 33.7% in 2016 [8]. This minimal decrease does not change the need for continued preventive efforts against the obesity epidemic in SA, especially with existing gender disparities in obesity prevalence: 39.5% vs. 29.5% in women vs. men, respectively [9].

A high body mass index (BMI) can be affected by the level of physical activity, diet, and certain environmental and social factors for which individuals have considerable influence. [9]. High BMI can put someone at an increased risk of mortality and multiple morbidities including diabetes mellitus, hypertension, dyslipidemia, cardiovascular diseases (CVD), and cancer [10,11,12]. Obesity is associated with an increased risk of death from all-cause mortality and CVD compared to people in the healthy weight range [13]. Notably, overweight and obesity tops the list of risk factors associated with disease burden in SA [14]. NCDs currently account for about 73% of Saudi deaths [8]. CVD, for which hypertension is a major risk factor, accounts for about 37% of deaths, making CVD the leading cause of death in SA [15]. Diabetes is in the top 10 causes of death (5% of total deaths) and disability (years lived with disability or YLDs). This makes it a significant contributor to the disease burden in SA [16]. The increasing burden of NCDs is not limited to population health; it also creates an increasing economic burden on the health system [12].

In 2017, SA adopted a unified excise tax on sugar-sweetened beverages [17]. Moreover, the government recently mandated that all food establishments label menus to show the meals’ calorie content to create greater public awareness of food choices [18]. Some studies showed an increasing trend of obesity prevalence in SA [19], but the WHO estimates tell a different story [8]. This discrepancy emphasizes the need for more updated and comprehensive data than what is already published. Furthermore, in light of the recent obesity preventive interventions, there is a need for an effective surveillance system to measure the impact of these interventions and monitor local progress. Most of the previous studies were based on convenience sampling using traditional data sources such as community surveys. 

An emerging and more comprehensive surveillance source of chronic diseases and other population health-related data is electronic medical records (EMR). The use of EMR data for chronic disease surveillance is encouraged because it can provide timely, geographically specific, and high-quality information [20,21]. Additionally, it was reported that once the EMR system is in place, the time and cost commitment for data extraction is minimal [22]. 

Therefore, an EMR system is a potentially cost-effective tool that can be used to bridge the understanding of the obesity epidemic in SA and its association with cardiometabolic diseases, including diabetes and hypertension. Awareness of the magnitude of the target population’s problem and characteristics is critical in targeting at-risk groups and designing effective population-based interventions. SA covers a wide geographic area, and its residents come from various cultural backgrounds. This study aimed to estimate the prevalence of overweight and obesity and their association with sociodemographic factors and morbidities in the central, eastern, and western provinces of the Kingdom.

## 2. Materials and Methods

This population-based study used the EMR system from the National Guard Health Affairs (NGHA) in SA. NGHA is a government entity that serves all employees of the national guard and their dependents. In addition, individuals with health insurance may also be seen privately through the business center. All healthcare services rendered via the business center (around 10% of all treated patients) are registered in the EMR. This network of five hospitals is in three regions of the Kingdom: central, western, and eastern regions. The network is estimated to serve around one million beneficiaries. Care is coordinated via a single EMR system known as BESTCare. This system was implemented in January 2016. The main medical city is in the capital of Riyadh, which is also the home for a large university for health sciences and a research center with branches in the eastern and western regions. In addition, there are several outpatient clinics distributed around the Kingdom that serve patients.

We included individuals aged 17 years or older who visited any outpatient clinic in the past four years (2016–2019) and had at least one reading of BMI. Measuring BMI is a standard part of clinical care during outpatient visits. This entails any visit to the outpatient clinic even for mild cases, such as flu. Therefore, it was likely that the information on body mass needed for calculating BMI was captured in most if not all of the underlying population. Data were captured from five settings providing healthcare to NGHA beneficiaries across the Kingdom. Patients were excluded if they died during the last hospitalization or after the latest outpatient visit. 

BMI was calculated automatically in the system using weight (in kilograms) divided by height (in meters squared). Subjects were then categorized as underweight (BMI < 18.5), normal (BMI = 18.5–24.9), overweight (BMI = 25–29.9), or obese (≥30) [5]. The following variables were obtained from the BESTCare system: age, gender, nationality, BMI, diabetes, hypertension, and cancer. If the patient attended an outpatient appointment or was hospitalized for any reason, a primary diagnosis was documented. Patients were classified as diabetic if the discharge diagnosis was diabetes. The same was true for hypertensive patients. The keywords “diabetes” and “hypertension” were used to query the electronic system to identify patients using the diagnosis option. In addition, a patient was identified as diabetic or hypertensive based on whether he or she was using any medication for that condition. For DM, the medications included alpha-glucosidase inhibitors, biguanides, thiazolidinediones, sulfonylureas, and meglitinides, while antihypertensive medication included beta blockers, ACE inhibitors, angiotensin II receptor blockers, calcium channel blockers, and adrenergic inhibitors and vasodilators [23,24]. There was no informed consent in this study since it was an observation study from medical charts. This study was reviewed and approved by the Institutional Review Board (IRB) of King Abdullah International Medical Research Center (KAIMRC) study number 19/189/R. 

### Statistical Analysis

Stata 15 and Excel for Mac were used in all analyses. Descriptive statistics by BMI category were calculated for various variables, and differences by demographic characteristics were evaluated using chi-squared tests. A *p*-value of <0.05 was considered statistically significant. In addition, differences in BMI categories were depicted across genders, regions, and nationalities.

To evaluate the association between obesity and diabetes or hypertension controlling for multiple factors, we constructed logistic regression models to calculate the odds ratios (ORs) and associated 95% confidence intervals. Normal weight individuals (BMI = 18.5–24.9) served as the reference category. Age was categorized into 17–25 as the reference and 26–45, 46–64, and 65 and older. Females and the central region were used as the reference groups for gender and region, respectively.

## 3. Results

The study initially identified 876,602 individuals. After excluding those younger than 17 years old, 616,092 individuals remained. Most of the study population were Saudi nationals, 573,698 (93.1%), and 338,724 (55%) of the overall population were females. The distribution of individuals in each BMI category was underweight = 33,332 (5.41%), normal weight = 162,100 (26.32%), overweight = 180,431 (29.30%), and obese = 239,913 (38.96%). Approximately 68% of the study population were either obese or overweight. Comorbidities, such as diabetes and hypertension, were found in 18.42% and 16.23% of individuals, respectively (Table 1).

**Figure 1:**
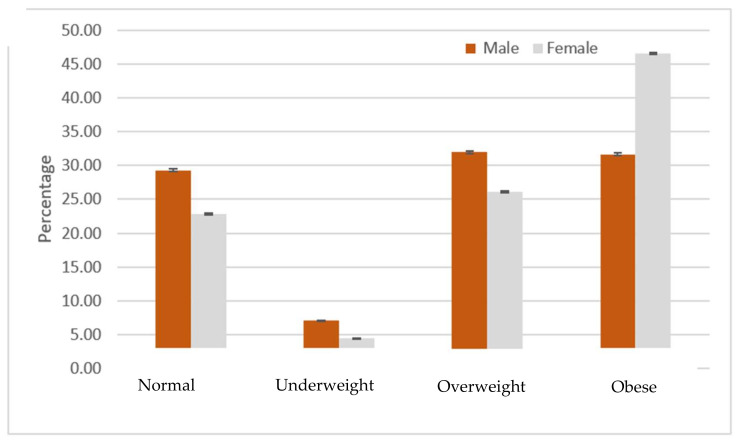
Prevalence of weight category by gender: Saudis (*n* = 573,698).

**Figure 2:**
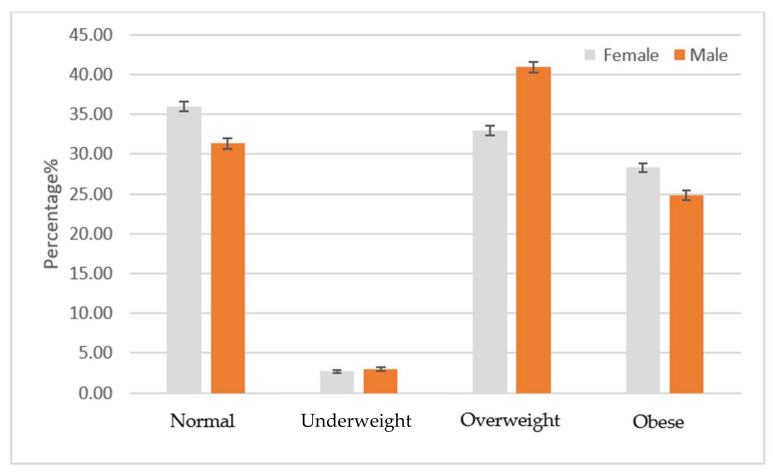
Prevalence of weight category by gender: Non-Saudis (*n* = 42,394).

Notably, a higher prevalence of obesity was observed among Saudi nationals than non-Saudis (39.9% vs. 26.7%, *p* < 0.01). Most non-Saudis fell in the overweight category (36.6%, Table 2). Obesity was more prevalent among older patients (56.6% in 46–64 and 45.9% in ≥65 age groups vs. 17.7% among < 26 years old, *p* < 0.01, Table 2). Females were more likely to be obese than males (45.3% vs. 31.2%, *p* < 0.01). This result was equally seen in Saudis and non-Saudis as depicted in Figure 1 and Figure 2.

BMI was independently associated with diabetes mellitus in the multivariable logistic analysis. Obese individuals were significantly more likely to have diabetes than those with normal weight (OR = 2.24, 95% CI = 2.19, 2.28, *p* < 0.01). Age was also a predictor of diabetes. Versus those younger than 26 years old, those older than 65 years were approximately 27-fold more likely to have diabetes (OR= 27.36, 95% CI = 26.32, 28.45, *p* < 0.01). Males were 6% more likely to be diagnosed with diabetes than females (OR = 1.06, 95% CI = 1.05, 1.08, *p* < 0.01). Western and eastern regions had 26% lower odds of diabetes than the central region (western: OR = 0.74, 95% CI = 0.72, 0.75, *p* < 0.01 and eastern OR = 0.90, 95% CI = 0.89, 0.92, *p* < 0.01, Table 3). 

An independent association was also observed between obesity and hypertension: obese patients were 2.1-fold more likely to have hypertension (*p*-value <0.01). Gender was also a significant predictor of hypertension. Males were 18% more likely to be diagnosed with hypertension than females (OR = 1.18, 95% CI = 1.16, 1.20, *p* < 0.01). When comparing Saudi regions to the central region, western and eastern areas were associated with a reduced likelihood of hypertension (western: OR = 0.62, 95% CI = 0.61, 0.63, *p* < 0.01 and eastern: OR = 0.89, 95% CI = 0.87, 0.92, *p* < 0.01, Table 3).

## 4. Discussion

This study found a significant association between obesity and a diagnosis of diabetes or hypertension. Our results revealed that around 45% of women in our sample were obese and 26% were overweight. Obesity prevalence was projected to rapidly increase between 1992 and 2022 from 12% to 41% among men and 21% to 78% among women [18]. Our findings underlined the major impact that obesity plays on NCDs and healthcare utilization and on population health. NCDs are currently responsible for around 73% of all deaths in the Kingdom [25]. If this burden continues, then it will likely play a devastating impact on the population in the next decade in the Kingdom. 

Using electronic records to examine the impact of interventions to reduce obesity and chronic conditions may help monitor and improve population health [26]. Other countries, such as the United States, explored using EMR to evaluate conditions, such as diabetes and hyperlipidemia [25].

Our results concurred with several other studies that assessed the burden of obesity, diabetes, and hypertension in Saudi Arabia. For example, Memish et al. showed in their 2013 national survey that only enrolled Saudis (ages 15 years or older) had an increase in the obesity burden among women (33% women vs. 24% men) [12]. A more recent study took a subsample of the 2013 Memish et al. data and found obesity to be associated with diabetes and hypertension [27]. However, both studies were limited to Saudis, and the latter one did not employ weighted analysis, which may have biased the results. Our large sample included both Saudi and non-Saudi populations and showed a wider extent of obesity prevalence (45% women vs. 31% men).

Our findings were also consistent with the Almajwal et al. study that showed an increased risk of diabetes and hypertension among the Saudi population relative to their BMI [28,29]. In addition, several previous regional cross-sectional studies indicated variations in obesity prevalence in the Kingdom [30,31]. Although these rates varied between regions due to limited sample size and differences in age groups, their findings consistently aligned with our findings on the higher obesity prevalence among women. Furthermore, our result on the obesity prevalence being higher among women was also similar to findings from a recently published study (PURE-Saudi) [31]. While our findings indicated that 16% of our study participants had hypertension and 18% had diabetes, the PURE-Saudi study showed a prevalence of 30% hypertension and 25% diabetes among participants. This might be explained by the older cohort in the PURE study or its limited representativeness.

Lifestyle has become more westernized and sedentary in the Kingdom during the last three decades, leading to an increased obesity prevalence among both men and women [32]. In particular, women were shown in our study and other previous research to have a higher prevalence of obesity than men [30]. A combination of social and policy factors may have led to this inequality. These factors showed that women are more prone to stay home. They also have limited access to culturally acceptable exercise activities, e.g., the high cost of female gyms relative to those for men [25]. 

Our findings have implications for both healthcare policies and population health initiatives and research funding. On the national level, these findings call for strengthening preventive care to reduce obesity in the Kingdom and to address inequality between men and women in terms of obesity burden and chronic disease management. Our findings can also be used to inform the modeling of future obesity burden and inform targeted awareness initiatives in the Kingdom. Finally, this study adds to the growing evidence that obesity and NCDs are increasing threats in the Kingdom.

The Saudi Vision 2030 is a strategic plan to effectively transform numerous sectors in the Kingdom, including healthcare [33]. Multiple initiatives under the Vision 2030 were recently implemented to reduce the burden of NCDs in the Kingdom and its risk factors, including obesity. For example, the Kingdom recently introduced a tax on carbonated drinks (50%), which was shown to be effective in lowering the consumption of carbonated beverages [16]. In addition, there is a new model of care being developed for the Saudi healthcare system as part of Vision 2030. This model prioritizes NCDs prevention and emphasizes the public health role in healthcare [11]. Addressing the inequalities between women and men is a critical indicator in Vision 2030. This comes alongside other public health initiatives to improve the quality of life in SA and promote women’s access to exercise facilities that are safe, affordable, and culturally acceptable [33]. Further studies need to assess the trend in women’s physical activity in light of the recent policies aimed to promote physical activity among women and evaluate the acceptability and efficacy of promoting home gyms in the Kingdom. 

Since 2016, the Kingdom has experienced rapid growth in home delivery of food via smartphone applications. Moreover, the Kingdom has also experienced both complete and partial lockdowns between March and June 2020 to mitigate the coronavirus disease 2019 (COVID-19) pandemic. Consequently, this inevitably limited physical activity [34]. These two factors were expected to contribute to the pre-existing obesity endemic in Saudi Arabia.

Epidemiological research remains vital to assess the current landscape of population health in the Kingdom. The use of EMR in our study provided an example of the potential use to monitor disease burden over time and across regions. As part of the Saudi Vision 2030, the government aims to implement EMR for every citizen or resident. Thus, linking existing records in the National Guard with other national sources will not pose a challenge and can further expand our understanding of the burden of obesity and associated conditions. Although Saudi Arabia’s research output on NCDs massively improved in the last decade, it still lags behind several countries in the region [35]. Our findings add to the existing body of literature and underscore the need to allocate funding to population health research, including the recently established Saudi National Institute of Health [36]. 

Our study had several strengths. First, the study included a large sample of diverse populations. To our knowledge, this was the largest cohort yet that aimed to determine the extent of the burden of obesity in Saudi Arabia. In fact, we do not know of any other study that used a population-based sample capturing more than half a million individuals in the Kingdom. Previous similar research was limited either to the Saudi population, a small sample size, or a specific region. In addition, BMI measurements were recorded during hospital visits by trained nurses, which improved the reliability of BMI measurements in our data. Finally, the use of a unified electronic system captured the latest data measured in terms of BMI or disease diagnosis for all patients. Every patient has a unique identifier that can identify him or her without duplication if treated in other clinics or regions. This helps future studies in terms of identifying targeted groups for prevention or intervention.

However, our study did have a few limitations. The data were based on visits to the healthcare facility, raising the possibility that the prevalence of obesity was overestimated versus the general population. This is because those who did not visit the hospital in a period of four years were not represented in our sample and likely to be healthier than those who visited the hospital or clinic. Still, even if that had occurred, it was expected that the magnitude of the bias was minimal because the NGHA provides healthcare to all military personnel, staff, and students who may show up at the clinic for a regular check-up. Second, some patients may have had a change in their BMI since their last visit due to nutritional programs, exercise, or other means of weight reduction. Nevertheless, it was unlikely that a drastic change occurred without any visit to our facility in which BMI was captured. Therefore, it is doubtful that this would affect our findings. Third, as the study population was part of the 3% of the overall Saudi population being either employees at the National Guard or their dependents, representativity towards the entire population cannot be assured. Fourth, only a very limited number of covariates were available in the data, and some essential factors were missing. For example, factors, such are diet or measures of physical activity, may have an impact of the burden of obesity or chronic conditions. Finally, the study was unable to differentiate between type 1 and type 2 diabetes. Only the latter is known to be associated with obesity, and the potential underestimation of association is possible.

## 5. Conclusions

In summary, this large population-based study showed a significant association between obesity, diabetes, and hypertension. The extent of obesity prevalence in the study population was high and more pronounced among women. These findings support the ongoing efforts to increase preventive measures and population health research. Future work is needed to continuously monitor the obesity trend and evaluate the efficacy of Vision 2030 obesity-related policies and initiatives. Using electronic records to examine obesity and chronic conditions may help monitor and improve population health.

## Figures and Tables

**Table 1:** Baseline characteristics of the patients treated in National Guard Hospitals from five regions (*n* = 616,092).

Characteristic Variables	*n* (%)
**Age categories**	
17–25	116,656 (18.94)
26–45	264,892 (43)
46–64	152,672 (24.79)
≥65	81,580 (13.25)
**Gender**	
Male	277,336 (45)
Female	338,724 (55)
**BMI/kg × m^−2^ categories**	
Underweight	33,324 (5.41)
Normal	162,100 (26.32)
Overweight	180,431 (29.30)
Obese	239,913 (38.96)
**Nationality**	
Saudi	573,698 (93.12)
Non-Saudi	42,102 (6.83)
**Region**	
Central	338,027 (54.89)
Western	178,204 (28.94)
Eastern	99,569 (16.17)
**Diabetes mellitus**	
Yes	113,409 (18.42)
No	502,391 (81.58)
**Hypertension**	
Yes	99,934 (16.23)
No	515,866 (83.77)

**Table 2:** Descriptive characteristics of the study population by BMI category.

Variables	Underweight *n* (%)	Normal *n* (%)	Overweight *n* (%)	Obese *n* (%)	*p*-Value
**Age categories**					
17–25	20,114(17.24)	51,716 (44.33)	24,098 (20.66)	20,728 (17.77)	<0.01 *
26–45	9713 (3.7)	74,762 (28.22)	85,091 (32.12)	95,326 (35.99)	
46–64	1422 (0.93)	19,262 (12.62)	45,564 (29.84)	86,424 (56.61)	
≥65	2083 (2.55)	16,370 (20.07)	25,689 (31.49)	37,438 (45.89)	
**Gender**					
Male	18,742 (6.76)	81,624 (29.45)	90,342 (32.59)	86,488 (31.20)	<0.01 *
Female	14,582 (4.31)	80,476 (23.77)	90,089 (26.61)	153,425 (45.32)	
**Nationality**					
Saudi	32,140 (5.6)	147,836(25.77)	165,048 (28.77)	228,674 (39.86)	<0.01 *
Non-Saudi	1192 (2.83)	14,274 (33.90)	15,394 (36.56)	11,242 (26.70)	
**Region**					
Central	17,489 (5.17)	88,254 (26.11)	99,079 (29.31)	133,205(39.41)	<0.01 *
Western	11,215 (6.29)	48,950 (27.47)	53,012 (29.75)	65,027 (36.49)	
Eastern	4628 (4.65)	24,906 (25.01)	28,351 (28.47)	41,684 (41.86)	
**Diabetes mellitus**					
Yes	1570 (1.38)	14,736 (12.99)	32,734 (28.86)	64,369 (56.76)	<0.01 *
No	31,762 (6.32)	147,374(29.33)	147,708 (29.40)	175,547(34.94)	
**Hypertension**					
Yes	1359 (1.36)	12,927 (12.94)	28,483 (28.50)	57,165 (57.20)	<0.01 *
No	31,973 (6.20)	149,183(28.92)	151,959 (29.46)	182,751(35.43)	

* Chi-squared tests.

**Table 3:** Logistic regression of the association between BMI and the likelihood of diabetes and hypertension.

	Adjusted Odds of Diabetes			Adjusted Odds of Hypertension		
Variables	Odds Ratio (OR)	95% CI	*p*-Value	Odds Ratio (OR)	95% CI	*p*-Value
**Age category**						
17–25	Reference					
26–45	2.34	2.26–2.43	<0.01 *	3.96	3.70–4.23	<0.01 *
46–64	13.37	12.87–13.89	<0.01 *	37.71	35.37–40.21	<0.01 *
≥65	27.37	26.32–28.45	<0.01 *	106.189	99.57–113.25	<0.01 *
**Gender**						
Female	Reference					
Male	1.06	1.05–1.08	<0.01 *	1.18	1.16–1.20	<0.01 *
**BMI**						
Normal weight	Reference					
Underweight	0.78	0.74–0.83	<0.01 *	0.86	0.81–0.92	<0.01 *
Overweight	1.56	1.52–1.59	<0.01 *	1.45	1.41–1.49	<0.01 *
Obese	2.24	2.19–2.29	<0.01 *	2.15	2.09–2.19	<0.01 *
**Region**						
Central	Reference					
Western	0.74	0.72–0.75	<0.01 *	0.62	0.61–0.63	<0.01 *
Eastern	0.90	0.88–0.92	<0.01 *	0.89	0.87–0.92	<0.01 *

* Chi-squared tests.

## Data Availability

All data are available from the corresponding author upon reasonable request.
